# A Machine Learning Framework for Prognostic Modeling in Stage III Colon Cancer

**DOI:** 10.3390/jcm15083091

**Published:** 2026-04-17

**Authors:** Rümeysa Sungur, Selin Aktürk Esen, Hilal Arslan, Sevil Uygun İlikhan, Hatice Rüveyda Akça, Efnan Algın, Öznur Bal, Şebnem Yaman, Doğan Uncu

**Affiliations:** 1 Department of Internal Medicine, Ankara Bilkent City Hospital, 06800 Ankara, Turkey; sevil.uygunilikhan@sbu.edu.tr; 2 Department of Medical Oncology, Ankara Bilkent City Hospital, 06800 Ankara, Turkey; drselin16@hotmail.com (S.A.E.);; 3 Department of Software Engineering, Ankara Yıldırım Beyazıt University, 06010 Ankara, Turkey; hilalarslan@aybu.edu.tr (H.A.); hruveydaakca106@gmail.com (H.R.A.)

**Keywords:** colon cancer, stage III colon cancer, prognosis, machine learning, survival analysis, artificial intelligence, risk factors

## Abstract

**Objective:** To evaluate overall survival and to identify clinical, pathological, and demographic factors associated with survival in patients with stage III colon cancer. **Methods:** This retrospective cross-sectional study included 452 patients with stage III colon cancer who were followed at Ankara Bilkent City Hospital between 2005 and 2025. Patient data, including age, sex, ECOG performance status, comorbidities, tumor characteristics, treatment-related toxicities, and recurrence, were analyzed using PASW Statistics 18.0 (SPSS Inc., Chicago, IL, USA). Kaplan–Meier and log-rank tests were used for survival analysis. Prognostic factors, survival, mortality, and recurrence predictions were evaluated using machine learning algorithms, including coarse tree, bagged trees, support vector machines, and k-nearest neighbors. Furthermore, an explainable artificial intelligence framework was incorporated to improve model transparency and reveal clinically meaningful feature contributions. Model performance was assessed using accuracy, sensitivity, specificity, and F-score. **Results:** According to statistical analyses, older age, ECOG performance score ≥ 2, stage IIIC disease, N2-level lymph node metastasis, and the presence of comorbidities—particularly diabetes mellitus—were significantly associated with worse survival (*p* < 0.05). Machine learning analyses identified key prognostic factors, including positive surgical margins, rash, mucositis, thrombocytopenia, number of chemotherapy cycles, pathological tumor subtype, diarrhea, age at diagnosis, and anemia. SHAP analysis further demonstrated that treatment-related variables, particularly surgical margin positivity and chemotherapy-associated toxicities, were among the most influential predictors of survival. Several machine learning models outperformed traditional statistical methods in predicting mortality and recurrence, with the highest accuracy observed in ensemble methods such as coarse tree (87%) and bagged trees. **Conclusions:** This study identifies key prognostic factors influencing survival in stage III colon cancer and demonstrates that machine learning-based approaches can complement conventional statistical methods. The integration of clinical and treatment-related variables may improve individualized risk stratification and support clinical decision-making. These findings may also guide future large-scale, multicenter, and prospective studies.

## 1. Introduction

Colorectal cancer is the third most common type of cancer in both men and women and ranks third among cancer-related deaths [1]. The development of colon cancer is influenced by genetic, environmental, and lifestyle factors. Accurately identifying prognostic factors that affect survival in patients with stage III disease is critical for treatment management. Although classical statistical analyses are frequently used to determine these factors, in recent years, artificial intelligence (AI) and machine learning methods have emerged with the potential to develop highly predictive models for complex data structures. These methods offer significant advantages in improving accuracy and enabling the development of personalized medical decision-support systems.

It has been demonstrated that deep learning-based systems can achieve expert-level accuracy in image analyses [2]. Similarly, AI models utilizing radiological and pathological data have been reported to predict cancer prognosis with high accuracy [3]. In the context of colon cancer, Wang et al. [4] showed that machine learning models using parameters derived from histopathological images achieved successful results in staging and survival prediction. Likewise, several studies have reported that machine learning models for survival prediction in colorectal cancer patients may outperform traditional regression-based approaches. These findings highlight the strong potential of AI algorithms in prognostic prediction and personalized treatment strategies for colon cancer.

The aim of the present study was to identify factors affecting survival in patients with stage III colon cancer using both classical statistical analyses and various machine learning algorithms, and to compare the performance of these approaches.

Unlike many previous studies that primarily focused on limited clinical or imaging-based datasets, the present study integrates a comprehensive set of clinical, pathological, and treatment-related variables, including chemotherapy-associated toxicities, in a relatively large real-world cohort of stage III colon cancer patients with long-term follow-up. Furthermore, this study provides a direct comparison between conventional statistical methods and multiple machine learning algorithms within the same dataset, enabling a comprehensive evaluation of their relative performance in survival and recurrence prediction.

To our knowledge, this is one of the few studies integrating chemotherapy-related toxicities into machine learning-based survival models in stage III colon cancer using a comprehensive real-world dataset and explainable artificial intelligence approaches. In addition to the use of machine learning techniques, the novelty of this study lies in several key aspects. First, this study integrates multidimensional data, including both baseline clinical and pathological features and detailed treatment-related variables, such as chemotherapy-associated toxicities, which are often underrepresented in prognostic modeling studies. Second, unlike many previous studies that rely solely on baseline prediction, our analysis incorporates longitudinal treatment-related data, reflecting the dynamic and cumulative nature of real-world clinical practice and providing a more holistic assessment of survival determinants. Third, this study evaluates and compares a wide range of machine learning algorithms alongside traditional statistical methods within the same dataset, enabling a direct and methodologically consistent comparison of their predictive performance. Finally, the application of explainable artificial intelligence techniques (SHAP) enhances model interpretability, bridging the gap between predictive performance and clinical applicability.

## 2. Materials and Methods

This retrospective cross-sectional study included a total of 452 patients aged 18 years and older who were diagnosed with stage III colon cancer and followed between 2005 and 2025 at the Department of Medical Oncology of Ankara Numune Training and Research Hospital and Ankara Bilkent City Hospital. Demographic, clinical, laboratory, and survival-related parameters were retrospectively collected from patient medical records. The variables included Eastern Cooperative Oncology Group Performance Status (ECOG-PS), age at diagnosis, sex, presence of comorbidities, tumor obstruction, tumor perforation, tumor localization, histological type, tumor grade, lymphovascular invasion, perineural invasion, pathological T stage (pT), pathological N stage (pN), overall disease stage, number of resected metastatic lymph nodes, surgical margin positivity, receipt of adjuvant chemotherapy, number of chemotherapy cycles, and the presence of chemotherapy-related adverse events (neuropathy, anemia, thrombocytopenia, leukopenia, neutropenia, nausea, vomiting, mucositis, diarrhea, and rash), as well as disease recurrence status. Overall survival (OS) was defined as the time from the date of diagnosis to death from any cause or the date of last follow-up. Survival data were obtained from the National Population Registry Database. The study was conducted as part of a medical specialty thesis at the University of Health Sciences.

Ethical approval for the study was obtained from the Academic Board of the University of Health Sciences and the Ethics Committee of the Republic of Türkiye Ministry of Health and Ankara Bilkent City Hospital (approval number: TABED 1/916/2025; date: 15 January 2025).

## 3. Treatment Protocols

As adjuvant chemotherapy, patients received one of the following regimens: FOLFOX, CAPOX, FUFA, or capecitabine monotherapy. In the FOLFOX regimen, oxaliplatin (85 mg/m^2^), leucovorin (400 mg/m^2^), bolus 5-fluorouracil (400 mg/m^2^), and continuous infusion 5-fluorouracil (2400 mg/m^2^) were administered every 14 days for a total of 12 cycles. In the CAPOX regimen, oxaliplatin (130 mg/m^2^) and capecitabine (2000 mg/m^2^) were administered every 21 days for six cycles. In the FUFA regimen, 5-fluorouracil (200–250 mg/m^2^ per day) was administered for seven consecutive days in each cycle, for a total of six cycles. For capecitabine monotherapy, oral capecitabine was administered at a dose of 1250 mg/m^2^ twice daily for 14 days, followed by a 7-day rest period, repeated every 21 days for eight cycles.

## 4. Statistical Analysis

All statistical analyses were performed using PASW Statistics 18.0 (SPSS Inc., Chicago, IL, USA). Descriptive statistics were presented as frequencies and percentages for categorical variables and as mean, standard deviation, median, 25th–75th percentile, minimum, and maximum values for continuous variables.

The normality of data distribution was assessed using the Kolmogorov–Smirnov and Shapiro–Wilk tests. Comparisons between categorical variables were conducted using the chi-square test when assumptions were met; otherwise, Fisher’s exact test or the Fisher–Freeman–Halton test was applied. For continuous variables that did not follow a normal distribution, the Mann–Whitney U test was used.

Multivariate analyses were performed using the Cox proportional hazards regression model, including variables that were clinically or statistically significant in univariate analyses. Both backward elimination and enter methods were applied.

Survival analyses were conducted using the Kaplan–Meier method, and comparisons between groups were performed using the log-rank test. In subgroup analyses, the adjusted log-rank test was used.

A two-sided *p* value < 0.05 was considered statistically significant.

## 5. Machine Learning–Based Data Analysis

Artificial intelligence analyses were conducted using Python (version 3.14.1) and MATLAB (version R2025b) software environments. Clinical, pathological, and laboratory data were utilized to identify factors influencing survival outcomes. To mitigate the risk of overfitting and ensure robust model evaluation, a 5-fold cross-validation strategy was adopted. The dataset was randomly divided into five equal parts. In each iteration, one subset was reserved for validation, while the remaining four subsets were used for training. This approach ensured that each subset was used for validation at least once. Importantly, all preprocessing steps, including feature selection procedures (ANOVA, chi-square test, Kruskal–Wallis test, and tree-based feature importance ranking), were performed exclusively within the training folds in each iteration. This ensured that the validation data remained completely unseen during model training and prevented information leakage. Model performance metrics, including accuracy, precision, recall, and F-score, were calculated for each fold and averaged to obtain overall performance estimates. This cross-validation framework provided a more reliable assessment of model generalizability and reduced the risk of overfitting.

During the feature selection process, analysis of variance (ANOVA), the chi-square test, the Kruskal–Wallis H test, and tree-based feature importance rankings were employed. Variables consistently identified as significant across these methods were included in the final modeling phase. The selected parameters included ECOG performance status, age at diagnosis, sex, presence of comorbidities, tumor obstruction, tumor perforation, tumor localization, histological type, tumor grade, lymphovascular invasion, perineural invasion, pathological T and N stages (pT, pN), overall stage, number of resected metastatic lymph nodes, surgical margin positivity, receipt of adjuvant chemotherapy, number of chemotherapy cycles, chemotherapy-related toxicities (neuropathy, anemia, thrombocytopenia, leukopenia, neutropenia, nausea, vomiting, mucositis, diarrhea, and rash), and recurrence status.

For variable selection, conventional statistical methods were initially applied. ANOVA was used to test for differences in mean values across groups [5]. The chi-square test was used to evaluate associations between categorical variables [6]. The Kruskal–Wallis H test was applied for nonparametric comparisons across multiple groups [7]. Additionally, tree-based feature importance scores were used to quantify the contribution of each variable to model performance [8].

Feature selection procedures were performed based on outcome variables related to survival and recurrence.

A variety of machine learning algorithms were implemented for model development and comparison. Linear and polynomial regression models were included to capture linear and nonlinear relationships among continuous variables [9]. Logistic regression was applied for binary classification tasks [10]. Support vector machines (SVMs) were used to construct optimal decision boundaries [11]. Decision trees were employed for their interpretability [8]. Ensemble methods, including random forest and gradient boosting, were used to improve predictive performance [12]. The k-nearest neighbors (k-NN) algorithm was used for classification based on proximity [13]. Naive Bayes was included as a probabilistic classifier [14]. Linear discriminant analysis (LDA) was applied to improve class separability [15]. Artificial neural networks (ANNs) were also implemented to capture complex nonlinear relationships [16]. Hyperparameters of the machine learning models were optimized using grid search within the cross-validation framework to ensure optimal model performance.

For all algorithms, performance metrics—including accuracy, precision, recall, and F-score—were calculated, and comparative evaluations were conducted.

To reduce the risk of overfitting and improve model robustness, multiple feature selection methods and machine learning algorithms were applied and compared. Model performance was evaluated using internal validation results obtained during the model development process. Variables consistently identified across different methods were included in the final models to enhance generalizability.

To enhance model interpretability, Shapley Additive Explanations (SHAP) analysis was performed to quantify the contribution of each feature to model predictions.

In this study, the primary aim of the machine learning models was not to provide baseline prognostic prediction at the time of diagnosis, but rather to explore overall survival patterns using comprehensive real-world data, including variables obtained during treatment and follow-up. Therefore, treatment-related variables, such as chemotherapy exposure, number of treatment cycles, and treatment-related toxicities, were intentionally included in the models. This approach was designed to reflect real-world clinical trajectories rather than to simulate a strictly baseline prediction model and, therefore, does not represent data leakage but rather a different modeling objective.

Missing or unknown values were handled using an imputation-based approach. For categorical variables, missing values were imputed using the mode (most frequent category), while for continuous variables, linear interpolation was applied. This approach was used to preserve the sample size and reduce potential bias associated with case exclusion.

Although an independent external validation dataset was not available due to the retrospective single-center design, the use of 5-fold cross-validation, with strict separation of training and validation processes in each iteration, was implemented to minimize overfitting and provide a more reliable estimation of model generalizability.

## 6. Results

The clinical, pathological, anatomical, demographic characteristics, and treatment modalities of the patients are summarized in Table 1. Missing or “unknown” values in the dataset were handled using an imputation-based approach rather than being treated as a separate category. For categorical variables, missing values were imputed using the mode (most frequent category), whereas for continuous variables, linear interpolation was applied. This strategy was adopted to preserve the overall sample size, prevent bias associated with case exclusion, and avoid introducing artificial categories that may negatively affect model performance and interpretability.

A total of 452 patients with stage III colon cancer were included in the study. The median age at diagnosis was 60 years (range, 28–88), and 283 patients (62.6%) were male. Good performance status (ECOG 0–1) was observed in 374 patients (82.7%). Comorbidities were present in 181 patients (40%), with the most common being diabetes mellitus in 81 patients (17.9%) and hypertension in 110 patients (24.3%). The most frequent tumor localization was the sigmoid colon, observed in 163 patients (36.1%). Tumor obstruction was detected in 111 patients (24.6%), and tumor perforation in 19 patients (4.2%). The most common pathological stage was stage IIIB, observed in 308 patients (68.1%), and the most frequent pathological T stage was pT3, observed in 343 patients (75.9%). Lymph node involvement at the pN1 level was observed in 336 patients (74.3%) (Table 1).

Regarding surgical lymph node dissection, 274 patients (60.6%) had ≥12 lymph nodes resected, while 53 patients (11.7%) had <12 lymph nodes removed, indicating inadequate lymph node dissection. Histopathological examinations revealed adenocarcinoma in 362 patients (80.1%) and mucinous carcinoma in 37 patients (8.2%). The most common tumor differentiation was moderate grade (*n* = 222, 49.1%). Lymphovascular invasion was detected in 201 patients (44.5%), perineural invasion in 121 patients (26.8%), and tumor budding in 86 patients (19%). Microsatellite status was evaluated in a subset of patients: microsatellite stable (MSS) disease was identified in 105 patients (23.2%), and microsatellite instability–high (MSI-H) in 16 patients (3.5%) (Table 1). As adjuvant therapy, the 6-month FOLFOX regimen was the most frequently administered protocol (*n* = 293, 64.8%). During treatment, dose reduction was required in 68 patients (15%), and treatment delay occurred in 106 patients (23.5%). Among treatment-related adverse events, the most common were thrombocytopenia (*n* = 111, 24.6%), neutropenia (*n* = 97, 21.5%), and neuropathy *(n* = 55, 12.2%) (Table 1). During the follow-up period, recurrence occurred in 100 patients (22.1%), of whom 59 cases (59%) presented with distant metastases. Positive surgical margins were reported in 5 patients (1.1%) (Table 1).

**Figure 1:**
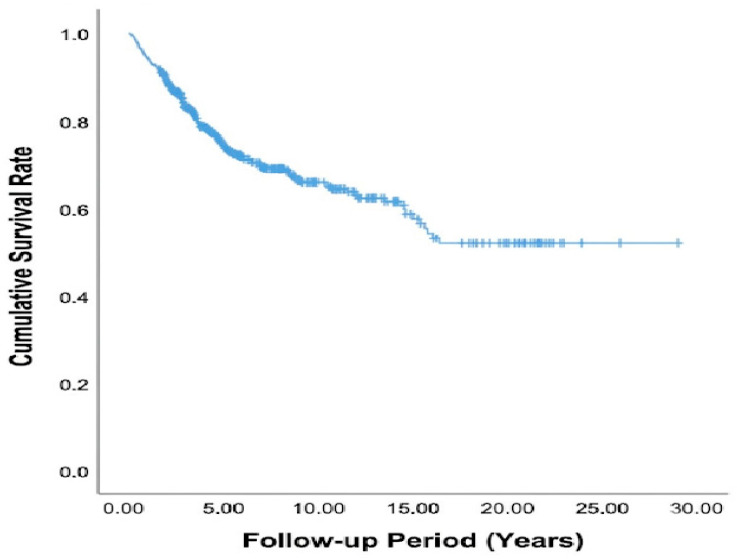
Kaplan–Meier curve showing median survival of the stage 3 colon cancer patient group.

The OS of patients diagnosed with stage III colorectal cancer was evaluated using the Kaplan–Meier method. As seen in Figure 1, the survival probability gradually decreased over time during the follow-up period; however, the median OS was not reached within the study duration. Based on the Kaplan–Meier survival estimates, the OS rates for stage III colon cancer were 87.29% at 2 years, 82.31% at 3 years, 72.32% at 5 years, 64.44% at 10 years, and 52.07% at 15 years.

**Figure 2:**
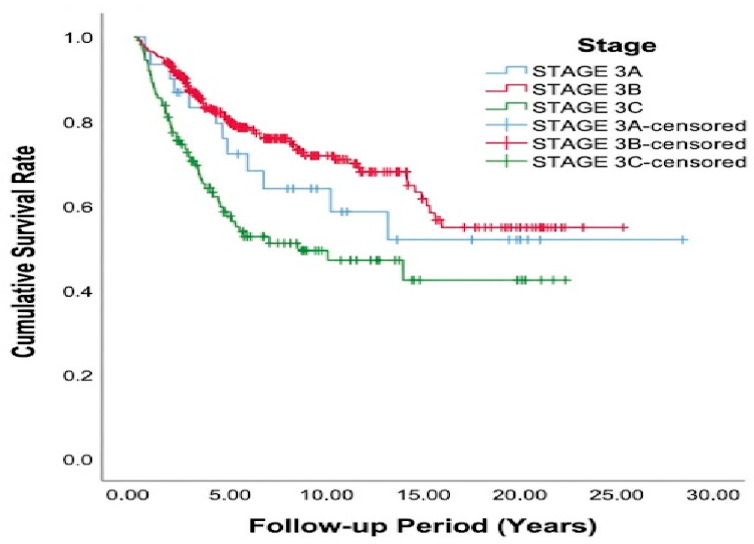
Kaplan–Meier curve showing median survival of the stage 3 colon cancer patient group according to stage.

Patients diagnosed with stage III colon cancer were further stratified into stage IIIA, IIIB, and IIIC subgroups according to the TNM classification, and OS was evaluated using the Kaplan–Meier method. As shown in Figure 2, the median OS was not reached in patients with stage IIIA or stage IIIB disease. In contrast, patients with stage IIIC disease demonstrated a significantly shorter median OS, calculated as 9.2 years (range, 2.4–15.9 years). A significant difference in OS was observed between stage IIIB and stage IIIC patients (*p* < 0.001). However, no significant difference was found between stage IIIA and stage IIIB (*p* = 0.401) or between stage IIIA and stage IIIC (*p* = 0.174). When OS rates were analyzed according to stage subgroup, the stage IIIB patients demonstrated the most favorable outcomes, with 5-year, 10-year, and 15-year OS rates of 79.0%, 71.4%, and 55.5%, respectively. In comparison, the stage IIIA patients had 5-year, 10-year, and 15-year OS rates of 72.7%, 59.2%, and 52.6%, respectively, while the stage IIIC patients exhibited the poorest survival outcomes, with corresponding rates of 54.6%, 47.7%, and 42.9%.

According to the univariate Cox regression analysis, several factors were significantly associated with OS. Increased age (HR: 1.025, 95% CI: 1.011–1.040, *p* = 0.001), an ECOG performance score of 2 (HR: 2.162, 95% CI: 1.186–3.940, *p* = 0.012), and the presence of comorbidities (HR: 1.639, 95% CI: 1.080–2.487, *p* = 0.020)—particularly diabetes mellitus (HR: 1.985, 95% CI: 1.268–3.107, *p* = 0.003)—were associated with higher mortality risk. Among the tumor-related parameters, stage IIIC disease (HR: 2.185, 95% CI: 1.539–3.101, *p* < 0.001), N2 lymph node involvement (HR: 2.003, 95% CI: 1.422–2.821, *p* < 0.001), tumor obstruction (HR: 1.678, 95% CI: 1.192–2.361, *p* = 0.003), mucinous histology (HR: 2.794, 95% CI: 1.782–7.832, *p* < 0.001), and perineural invasion (HR: 2.138, 95% CI: 1.429–3.198, *p* < 0.001) were identified as adverse prognostic indicators. Regarding the treatment-related variables, adjuvant capecitabine therapy (HR: 3.471, 95% CI: 1.056–11.406, *p* = 0.040) was associated with increased mortality risk. Recurrence was a particularly strong prognostic factor (HR: 6.715, 95% CI: 4.522–9.970, *p* < 0.001), especially distant metastatic recurrence (HR: 2.715, 95% CI: 1.448–5.092, *p* = 0.002). Among the chemotherapy-related adverse effects, neuropathy (HR: 2.070, 95% CI: 1.193–3.590, *p* = 0.010), anemia (HR: 2.852, 95% CI: 1.648–4.035, *p* < 0.001), neutropenia (HR: 1.816, 95% CI: 1.127–2.926, *p* = 0.014), nausea (HR: 2.605, 95% CI: 1.502–4.517, *p* = 0.001), vomiting (HR: 2.638, 95% CI: 1.395–4.990, *p* = 0.003), and diarrhea (HR: 3.772, 95% CI: 2.062–6.900, *p* < 0.001) were also identified as variables significantly increasing mortality risk (Table 2).

In the multivariate Cox regression analysis, stage IIIC disease (HR: 2.659, 95% CI: 1.124–6.293, *p* = 0.026), tumor obstruction (HR: 2.896, 95% CI: 1.265–6.627, *p* = 0.012), and perineural invasion (HR: 3.131, 95% CI: 1.334–7.346, *p* = 0.009) were identified as independent prognostic factors for OS (Table 2).

Table 3 presents the average performance metrics of various machine learning models predicting overall survival (OS) at different time points in stage III colorectal cancer subgroups (IIIA, IIIB, and IIIC), as well as in the entire cohort. The average performance values shown in Table 3 were calculated based on the results obtained from all machine learning models evaluated in Table 4 and Table 5. Although feature selection methods were applied, they resulted in only minimal changes in performance; therefore, models using the full set of variables were used for the final evaluation. Machine learning analyses provided OS predictions at 2-, 3-, 5-, 10-, and 15-year intervals across all groups. Notably, the highest performance values were observed for 15-year OS prediction. At this time point, mean precision ranged from 91% to 97%, mean recall reached 99%, mean F-score ranged from 95% to 98%, and mean accuracy ranged from 91% to 97% (Table 3).

Table 4 presents the accuracy rates of various machine learning methods for predicting mortality. Without feature selection, using all 29 variables (including ECOG-PS, age at diagnosis, sex, comorbidities, tumor obstruction, tumor perforation, tumor location, histology, grade, lymphovascular invasion, perineural invasion, pT, pN, stage, number of metastatic lymph nodes removed, surgical margin positivity, administered adjuvant chemotherapy, number of chemotherapy cycles, presence of neuropathy, anemia, thrombocytopenia, leukopenia, neutropenia, nausea, vomiting, mucositis, diarrhea, rash, and recurrence status), accuracy rates ranged from 69% to 81%, with the highest accuracy of 81% achieved by the coarse tree method. When feature selection was applied using ANOVA, three variables (number of chemotherapy cycles, tumor location, and rash) were excluded, and the best accuracy remained 81%, again with the coarse tree algorithm. Using the chi-squared test for feature selection, two variables (rash and mucositis) were excluded, and the highest accuracy of 81% was obtained with the bagged trees method. With the Kruskal–Wallis method, tumor location and rash were excluded, and the top accuracy of 80% was also achieved by the bagged trees algorithm (Table 4).

Table 5 displays the accuracy rates of various machine learning methods used to predict recurrence development. Without applying feature selection, 28 variables (including ECOG-PS, age at diagnosis, sex, presence of comorbidities, tumor obstruction, tumor perforation, tumor location, histology, grade, lymphovascular invasion, perineural invasion, pT, pN, stage, number of metastatic lymph nodes removed, surgical margin positivity, administered adjuvant chemotherapy, number of chemotherapy cycles, presence of neuropathy, anemia, thrombocytopenia, leukopenia, neutropenia, nausea, vomiting, mucositis, diarrhea, and rash) were included, resulting in accuracy rates ranging from 73% to 84%. The highest accuracy of 84% was achieved by the coarse tree method. Following feature selection by ANOVA, the variables mucositis, surgical margin positivity, and neuropathy were excluded; with the remaining 25 features, accuracy ranged between 72% and 82%. The best performances, with 82% accuracy, were obtained using the coarse tree, medium Gaussian SVM, boosted trees, and bagged trees algorithms. Using the chi-squared test for feature selection, four variables (adjuvant chemotherapy, mucositis, surgical margin positivity, and neuropathy) were excluded, and the highest accuracy of 84% was achieved with the boosted trees method. The Kruskal–Wallis method excluded seven variables (grade, thrombocytopenia, neutropenia, neuropathy, surgical margin positivity, ECOG-PS, and mucositis); with the remaining 21 features, the best accuracy of 83% was achieved by the coarse tree, efficient linear SVM, linear SVM, medium Gaussian SVM, and boosted trees algorithms (Table 5).

To enhance interpretability and address the “black-box” nature of machine learning models, SHAP (Shapley Additive Explanations) was used to quantify feature contributions. SHAP analysis demonstrated that treatment-related variables, including rash, positive surgical margin, mucositis, and thrombocytopenia, had the highest importance scores, highlighting the significant role of treatment-related factors in survival prediction.

**Figure 3:**
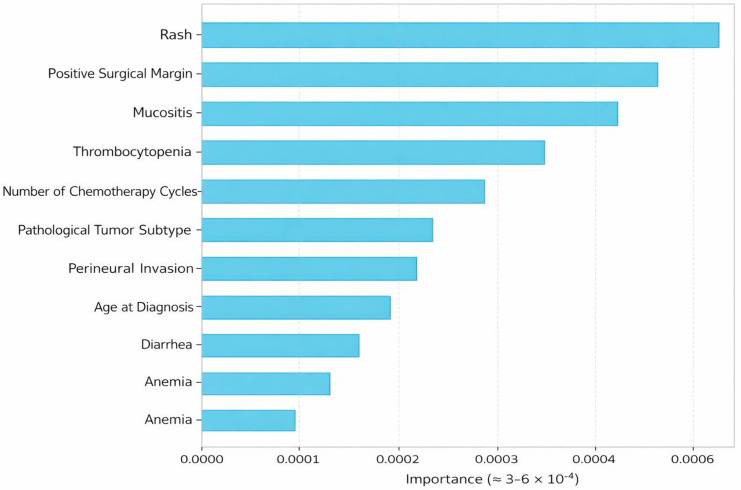
The impact of parameters affecting survival in colon cancer.

Figure 3 presents the top 10 most important features identified by SHAP. These findings support that the proposed models not only achieve strong predictive performance but also provide clinically interpretable insights into the factors influencing survival outcomes.

## 7. Discussion

In our study, the median age at diagnosis was 60 years. This value is close to that for the series reported by Dan et al. in China (61.5 years) [17] and the age distribution in the American Cancer Society’s 2023 report [18]. Although an increase in incidence under the age of 50 has been observed in recent years, the higher mean age in our study is expected since only stage III colon cancer patients were included. Additionally, advanced age was found to have a negative impact on survival, which is consistent with findings reported in the literature [19]. The association of advanced age with poor prognosis may be explained by an increased burden of comorbidities, limitations in treatment options, and decreased chemotherapy tolerance. The proportion of male patients was 62.6%, similar to the male predominance reported in large series from different geographic regions [18]. The tumor was most frequently located in the sigmoid colon (36.1%), showing left-sided predominance as observed in the large series by Warschkow et al. [20]. Examination of the pathological subtypes of the patients revealed that adenocarcinoma was the most common histology, consistent with the literature and our study [21]. Furthermore, mucinous histology emerged as a factor worsening survival; meta-analyses have shown that this subtype is associated with poor chemotherapy response [22]. The recurrence rate in the present study was 22.1%, which is close to the 20–30% range reported in the literature [23]. However, especially distant metastatic recurrence significantly reduced survival compared to local recurrence. This finding is consistent with studies highlighting the critical role of distant metastasis development in prognosis [24], underscoring the importance of early monitoring for distant metastasis.

In the multivariate analyses, stage IIIC, obstruction, and perineural invasion (PNI) were identified as independent prognostic factors. In the Kaplan–Meier analysis of the stage III colon cancer subgroups, median survival time was significantly shorter in the patients with stage IIIC disease. Similarly, large multicenter studies have shown that stage IIIC disease carries the worst prognosis in terms of survival [25]. This difference can be explained by more advanced nodal involvement and the higher frequency of micrometastatic spread in stage IIIC patients. Greene et al. [25] reported five-year survival rates of 59.8%, 42%, and 27.3% for stages IIIA, IIIB, and IIIC, respectively. In our series, these rates were 72.7%, 79%, and 54.6%, respectively, with higher survival in the IIIB group compared to IIIA, attributed to sample imbalance. In our study, the rate of obstruction at diagnosis was 24.6%, and it was found to be an independent factor negatively affecting survival in the multivariate analyses. As reported by Chen et al. [26], obstruction and perforation are factors that adversely affect prognosis; this finding aligns with our study’s identification of obstruction as an independent risk factor. The lack of significance for tumor perforation in our study may have been related to the small number of cases. In the present study, the rates of PNI and lymphovascular invasion (LVI) were 32.6% and 65.7%, respectively, with PNI identified as an independent prognostic factor increasing mortality risk. Yüksel et al. [27] and Huh et al. [28] similarly reported PNI as a strong determinant of survival. In patients presenting with these three factors, treatment regimens might be adjusted from 3-month to 6-month courses or from single-agent to multi-agent therapies. This trio can provide a practical risk scale for guiding adjuvant treatment intensity and follow-up frequency in stage 3 patients.

In our study, there was partial overlap between the prognostic factors identified through the classical statistical analyses (stage IIIC disease, tumor obstruction, and presence of PNI) and the variables highlighted in the machine learning-based analyses (positive surgical margin, mucositis, rash, thrombocytopenia, number of chemotherapy cycles, tumor subtype, diarrhea, age at diagnosis, and anemia). In the AI models, especially positive surgical margin and mucositis were found to have high importance scores in survival prediction. In the study by Peterson et al. [29], the presence of mucositis was associated with chemotherapy dose interruptions and shorter survival time. Additionally, the significance of the number of chemotherapy cycles aligns with data reporting better survival in patients receiving more than six cycles of treatment [30]. The most common chemotherapy-related side effects were thrombocytopenia, neutropenia, neuropathy, and anemia. It is thought that these toxicities indirectly affect survival by disrupting treatment continuity or reducing dose intensity rather than directly impacting survival. In particular, gastrointestinal side effects (nausea, vomiting, and diarrhea) can limit treatment intensity and adversely affect long-term survival. Our findings generally align with those reported by Sargent et al., although some differences in rates were observed due to the limitations of retrospective data [31]. In addition, SHAP (Shapley Additive Explanations) analysis provided further insight into the contribution of individual variables to model predictions. Consistent with our findings, treatment-related variables such as surgical margin positivity, mucositis, rash, and thrombocytopenia were identified as key contributors to survival outcomes. This approach enhances model interpretability and supports its potential clinical applicability.

The coarse tree and bagged trees algorithms achieved the highest accuracy, reaching up to 95%, and outperformed the traditional regression analyses in predicting recurrence and mortality. Consistent with our findings, previous studies employing random forest and SVM models have reported high accuracy in long-term survival prediction [32,33]. Seven et al. [34] demonstrated that machine learning techniques could reliably estimate 6-month, 1-year, 2-year, and 3-year overall survival probabilities across all stages of hepatocellular carcinoma, with the coarse decision tree model achieving 84.5% accuracy. Among the machine learning approaches, SVMs and ANNs are particularly favored for survival prediction in oncology [35]. Kourou et al. [35] showed that ANNs, Bayesian networks, SVMs, and decision trees outperformed conventional statistical methods in predicting survival and chemotherapy-related toxicities in lung cancer patients. Similarly, Abbas et al. [36] reported that neural networks achieved 65–97.5% accuracy in predicting recurrence among patients with non-muscle-invasive bladder cancer, outperforming SVMs, which averaged around 75%. These findings emphasize the clinical potential of intelligent systems capable of early risk stratification, particularly for malignancies with high recurrence rates. A systematic review by Wu et al. [37] on hepatocellular carcinoma confirmed that AI-based recurrence prediction models achieved moderate-to-high accuracy in estimating posttreatment relapse risk. In gastric cancer, comparative analyses of coarse tree and random forest models indicated that, although random forest demonstrated superior accuracy, the coarse tree model offered advantages in clinical implementation due to its simplicity and interpretability—underscoring the importance of balancing model complexity with practical usability [38].

Although the machine learning algorithms used in this study are not newly developed methods, the novelty of our work lies in the comprehensive integration of multidimensional real-world clinical data, including treatment-related toxicities, and in the use of explainable artificial intelligence techniques to enhance model interpretability. Furthermore, unlike many previous studies that rely solely on baseline variables, our approach incorporates longitudinal treatment-related information, allowing for a more realistic representation of clinical practice and patient trajectories.

This study has several limitations. First, its retrospective design may introduce selection bias. Missing data were handled using an imputation-based approach, which may influence the generalizability of the findings. Moreover, the use of imputation methods may have introduced bias, particularly if the data were not missing at random. Although this approach was chosen to preserve sample size and avoid case exclusion, its potential impact on model performance and estimates should be considered when interpreting the results. In addition, categorical variables with multiple levels were interpreted relative to the reference category, and global statistical comparisons across all levels were not performed; therefore, the results for variables such as ECOG performance status should be interpreted with caution.

The inclusion of treatment-related variables may limit the direct applicability of the model for baseline prognostic prediction at diagnosis; however, this design was intentional and aimed to better reflect real-world clinical processes and longitudinal patient management. Another limitation is the lack of external validation using an independent dataset. Although internal validation was performed using 5-fold cross-validation, external validation in multicenter cohorts would further strengthen the generalizability and robustness of the proposed models. Future multicenter prospective studies incorporating external validation cohorts are warranted to confirm the generalizability and clinical applicability of the proposed models.

The direct comparison of multiple machine learning models with traditional statistical approaches within the same dataset provides practical insights into their relative strengths and potential clinical applicability in routine oncology practice. In line with these findings, the strong performance of the coarse tree and bagged trees models in our study suggests their potential utility as reliable clinical decision-support tools in stage III colon cancer management.

## 8. Conclusions

This study underscores the prognostic relevance of both clinical and treatment-related factors in stage III colon cancer while demonstrating that machine learning-based models can surpass traditional statistical approaches in survival prediction. By integrating complex, multidimensional clinical and pathological data, these models offer a robust framework for individualized risk stratification and tailored treatment planning. The incorporation of such AI-driven approaches into oncological workflows may ultimately enhance prognostic accuracy and optimize therapeutic decision-making. The use of explainable artificial intelligence approaches, such as SHAP, further supports the interpretability and clinical applicability of these models. To ensure clinical translation and generalizability, future large-scale, multicenter prospective studies are essential to validate these findings and support the adoption of machine learning-assisted decision-support systems in routine clinical practice.

## Figures and Tables

**Table 1:** Clinical, pathological, anatomical, demographic characteristics, treatment modalities, and outcomes of patients diagnosed with stage 3 colon cancer.

		*N*	
Age at Diagnosis, Mean ± SD Median (Q1–Q3) (Min-Max)		452	58.77 ± 12.10 60.00 (50.00–67.00) (28.00–88.00)
Preoperative CEA Level, Mean ± SD Median (Q1–Q3) (Min-Max)		15	41.09 ± 80.42 13.00 (2.00–26.00) (1.00–310.00)
Preoperative Ca 19-9 Level, Mean ± SD Median (Q1–Q3) (Min-Max)		20	44.57 ± 60.93 17.48 (10.50–58.00) (0.08–250.00)
Number of CT Applications, Mean ± SD Median (Q1–Q3) (Min-Max)		380	10.45 ± 2.79 12.00 (8.50–12.00) (2.00–16.00)
Sex, *n* (%)	Male	452	283 (62.6)
	Female		169 (37.4)
ECOG-PS, *n* (%)	0	452	147 (32.5)
	1		227 (50.2)
	2		30 (6.6)
	3		6 (1.3)
	Unknown		42 (9.3)
Comorbidity, *n* (%)	No	452	199 (44.0)
	Yes		181 (40.0)
	Unknown		72 (16.0)
DM, *n* (%)	No	452	299 (66.2)
	Yes		81 (17.9)
	Unknown		72 (15.9)
HT, *n* (%)	No	452	270 (59.7)
	Yes		110 (24.3)
	Unknown		72 (16.0)
CAD, *n* (%)	No	452	323 (71.5)
	Yes		57 (12.6)
	Unknown		72 (15.9)
Other, *n* (%)	No	452	364 (80.5)
	Yes		16 (3.5)
	Unknown		72 (16)
Tumor Localization, *n* (%)	Sigmoid	452	163 (36.1)
	Descending Colon		57 (12.6)
	Transverse Colon		24 (5.3)
	Ascending Colon		117 (25.9)
	Unknown		91 (20.1)
Stage, *n* (%)	Stage 3A	452	31 (6.9)
	Stage 3B		308 (68.1)
	Stage 3C		113 (25.0)
pT, *n* (%)	T1	452	3 (0.7)
	T2		27 (6.0)
	T3		343 (75.9)
	T4		79 (17.5)
pN, *n* (%)	N1 = 1–3 Ln	452	336 (74.3)
	N2 ≥ 4 Ln		116 (25.7)
Removed LN, *n* (%)	<12	452	53 (11.7)
	≥12		274 (60.6)
	Unknown		125 (27.7)
Tumor Obstruction, *n* (%)	Yes	452	111 (24.6)
	No		283 (62.6)
	Unknown		58 (12.8)
Tumor Perforation, *n* (%)	Yes	452	19 (4.2)
	No		375 (83.0)
	Unknown		58 (12.8)
Histology, *n* (%)	Adenocarcinoma	452	362 (80.1)
	Signet Ring Cell Carcinoma		4 (0.9)
	Mucinous Carcinoma		37 (8.2)
	Unknown		49 (10.8)
Differentiation Grade, *n* (%)	Well Differentiated/Grade 1	452	111 (24.6)
	Moderate Differentiated/Grade 2		222 (49.1)
	Poorly Differentiated/Grade 3		28 (6.2)
	Undifferentiated/Grade 4		1 (0.2)
	Unknown		90 (19.9)
Lymphovascular Invasion, *n* (%)	Yes	452	201 (44.5)
	No		129 (28.5)
	Unknown		122 (27.0)
Perineural Invasion, *n* (%)	Yes	452	121 (26.8)
	No		210 (46.5)
	Unknown		121 (26.8)
Tumor Budding, *n* (%)	No	452	29 (6.4)
	Yes		86 (19.0)
	Unknown		337 (74.6)
Microsatellite Instability, *n* (%)	MSS	452	105 (23.2)
	MSI-H		16 (3.5)
	Unknown		331 (73.2)
Adjuvant Chemotherapy, *n* (%)	Drug-free follow-up	452	19 (4.2)
	6 Months FUFA		31 (6.9)
	6 Months Capecitabine		11 (2.4)
	6 Months FOLFOX		293 (64.8)
	6 Months CAPOX		35 (7.7)
	3 Months CAPOX		23 (5.1)
	3 Months FOLFOX		8 (1.8)
	Unknown		32 (7.1)
Dose Reduction, *n* (%)	Yes	452	68 (15.0)
	No		208 (46.0)
	Unknown		176 (40.0)
Dose Delay, *n* (%)	Yes	452	106 (23.5)
	No		176 (38.9)
	Unknown		170 (37.6)
Relapse, *n* (%)	Yes	452	100 (22.1)
	No		277 (61.3)
	Unknown		75 (16.6)
If there is recurrence, *n* (%)	Local Recurrence	100	30 (30.0)
	Distant Metastasis		59 (59.0)
	Unknown		11 (11.0)
Surgical Border, *n* (%)	Positive	452	5 (1.1)
	Negative		392 (86.7)
	Unknown		55 (12.2)
Neuropathy, *n* (%)	Yes	452	55 (12.2)
	No		221 (48.9)
	Unknown		176 (38.9)
Anemia, *n* (%)	Yes	452	45 (10.0)
	No		237 (52.4)
	Unknown		170 (37.6)
Thrombocytopenia, *n* (%)	Yes	452	111 (24.6)
	No		164 (36.3)
	Unknown		177 (39.1)
Leukopenia, *n* (%)	Yes	452	42 (9.3)
	No		224 (49.6)
	Unknown		186 (41.1)
Neutropenia, *n* (%)	Yes	452	97 (21.5)
	No		195 (43.1)
	Unknown		160 (35.4)
Nausea, *n* (%)	Yes	452	36 (8.0)
	No		245 (54.2)
	Unknown		171 (37.8)
Vomiting, *n* (%)	Yes	452	25 (5.5)
	No		252 (55.8)
	Unknown		175 (38.7)
Mucositis, *n* (%)	Yes	452	4 (0.9)
	No		256 (56.6)
	Unknown		192 (42.5)
Diarrhea, *n* (%)	Yes	452	28 (6.2)
	No		246 (54.4)
	Unknown		178 (39.4)
Debris, *n* (%)	Yes	452	8 (1.8)
	No		236 (52.2)
	Unknown		208 (46.0)

Ca19-9: Carbohydrate antigen 19-9, CAPOX: Oxaliplatin and capecitabine, CEA: Carcinoembryonic antigen, DM: Diabetes Mellitus, ECOG PS: Eastern Cooperative Oncology Group Performance Score, FOLFOX: Folinic acid + Fluorouracil + Oxaliplatin, FUFA: 5-fluorourosyl + calcium folinate, HT: Hypertension, CAD: Coronary Artery Disease, CT: Chemotherapy, LN: Lymph Node.

**Table 2:** Univariate and multivariate Cox regression analysis for overall survival.

	Univariate Analysis		Multivariate Analysis *	
	HR (95% CI)	*p*	HR (95% CI)	*p*
Sex (Female)	0.838 (0.591–1.19)	0.324		
**Age at Diagnosis**	1.025 (1.011–1.04)	**0.001**		
Preoperative CEA Level	1.181 (0.857–1.627)	0.309		
Preoperative CA19-9 Level	1.022 (0.967–1.082)	0.439		
**ECOGPS (0) (Reference)**	-	**0.035**		
ECOGPS (1)	1.39 (0.937–2.061)	0.102		
**ECOGPS (2)**	2.162 (1.186–3.94)	**0.012**		
**Comorbidity**	1.639 (1.08–2.487)	**0.020**		
**DM**	1.985 (1.268–3.107)	**0.003**		
HT	1.389 (0.887–2.175)	0.151		
CAD	1.652 (0.984–2.774)	0.058		
Other	1.74 (0.76–3.985)	0.190		
Tumor Localization (Sigmoid) (Reference)	-	0.419		
Tumor Localization (Descending Colon)	1.565 (0.897–2.729)	0.114		
Tumor Localization (Transverse Colon)	0.869 (0.309–2.443)	0.790		
Tumor Localization (Ascending Colon)	1.173 (0.719–1.913)	0.523		
**Stage (3C)**	2.185 (1.539–3.101)	**<0.001**	2.659 (1.124–6.293)	**0.026**
pT (T1) (Reference)	-	0.275		
pT (T2)	0.796 (0.102–6.232)	0.828		
pT (T3)	0.789 (0.11–5.665)	0.814		
pT (T4)	1.181 (0.161–8.653)	0.870		
**pN (N2 ≥ 4 LN)**	2.003 (1.422–2.821)	**<0.001**		
Removed LN (≥12)	1.133 (0.636–2.017)	0.671		
**Tumor Obstruction**	1.678 (1.192–2.361)	**0.003**	2.896 (1.265–6.627)	**0.012**
Tumor Perforation	1.443 (0.733–2.841)	0.289		
**Histology (Adenocarcinoma) (Reference)**	-	**<0.001**		
Histology (Signet Ring Cell Carcinoma)	1.091 (0.152–7.832)	0.931		
**Histology (Mucinous Carcinoma)**	2.794 (1.782–4.381)	**<0.001**		
Differentiation Grade (Well Differentiated/Grade 1) (Reference)	-	0.467		
Differentiation Grade (Medium Differentiated/Grade 2)	0.911 (0.617–1.345)	0.639		
Differentiation Grade (Poorly Differentiated/Grade 3)	1.338 (0.704–2.542)	0.375		
Lymphovascular Invasion	0.821 (0.551–1.223)	0.332		
**Perineural Invasion**	2.138 (1.429–3.198)	**<0.001**	3.131 (1.334–7.346)	**0.009**
Tumor Budding	1.814 (0.466–7.059)	0.390		
Microsatellite Instability (MSI-H)	0.966 (0.285–3.266)	0.955		
**Adjuvant Chemotherapy (Drug-Free Follow-Up) (Reference)**	-	**0.026**		
Adjuvant Chemotherapy (6 Months FUFA)	0.823 (0.28–2.422)	0.724		
**Adjuvant Chemotherapy (6 Months CAPECITABINE)**	3.471 (1.056–11.406)	**0.040**		
Adjuvant Chemotherapy (6 Months FOLFOX)	1.022 (0.415–2.514)	0.963		
Adjuvant Chemotherapy (6 Months CAPOX)	1.049 (0.343–3.208)	0.933		
Adjuvant Chemotherapy (3 Months CAPOX (XELOX))	2.248 (0.751–6.727)	0.148		
Adjuvant Chemotherapy (3 Months FOLFOX)	0.447 (0.052–3.824)	0.462		
Dose Reduction	0.730 (0.453–1.179)	0.198		
Dose Delay	0.670 (0.432–1.040)	0.074		
**Presence of Relapse**	6.715 (4.522–9.97)	**<0.001**		
**If there is recurrence (Distant Metastasis)**	2.715 (1.448–5.092)	**0.002**		
Surgical Border (Positive)	1.131 (0.279–4.575)	0.863		
**Neuropathy**	2.070 (1.193–3.590)	**0.010**		
**Anemia**	2.852 (1.648–4.935)	**<0.001**		
Thrombocytopenia	1.255 (0.727–2.167)	0.416		
Leukopenia	0.829 (0.353–1.945)	0.666		
**Neutropenia**	1.816 (1.127–2.926)	**0.014**		
**Nausea**	2.605 (1.502–4.517)	**0.001**		
**Vomiting**	2.638 (1.395–4.990)	**0.003**		
Mucositis	3.492 (0.475–25.687)	0.219		
**Diarrhea**	3.772 (2.062–6.900)	**<0.001**		
Debris	1.242 (0.301–5.118)	0.764		

Note: Differentiation Grade “Undifferentiated/Grade 4” and ECOG PS “3” Stage “3A” were not included in the analysis due to an insufficient number of patients. * Multivariate: Cox regression analysis was performed using the enter method. Ca19-9: Carbohydrate antigen 19-9, CAPOX: Oxaliplatin and capecitabine, CEA: Carcinoembryonic antigen, DM: Diabetes Mellitus, ECOG PS: Eastern Cooperative Oncology Group Performance Score, FOLFOX: Folinic acid + Fluorouracil + Oxaliplatin, FUFA: 5-fluorourosyl + calcium folinate, HT: Hypertension, CAD: Coronary Artery Disease, LN: Lymph Node.

**Table 3:** Average accuracy, average recall, average F-measure, and average accuracy values of the machine learning method used to predict patients’ overall survival.

	2-Year OS	3-Year OS	5-Year OS	10-Year OS	15-Year OS
Mean Precision					
All patients	0.73	0.79	0.83	0.89	**0.96**
Stage 3A	0.50	0.64	0.82	0.83	**0.91**
Stage 3B	0.55	0.78	0.86	0.93	**0.95**
Stage 3C	0.69	0.78	0.81	0.93	**0.97**
Mean Recall					
All patients	0.49	0.67	0.87	0.99	**0.99**
Stage 3A	0.42	0.59	0.86	0.86	**0.99**
Stage 3B	0.40	0.60	0.88	0.98	**0.99**
Stage 3C	0.52	0.72	0.94	0.98	**0.99**
Mean F-measure					
All patients	0.52	0.69	0.85	0.94	**0.98**
Stage 3A	0.44	0.60	0.84	0.84	**0.95**
Stage 3B	0.44	0.63	0.87	0.95	**0.97**
Stage 3C	0.53	0.74	0.86	0.95	**0.98**
Mean Accuracy					
All patients	0.87	0.85	0.84	0.89	**0.95**
Stage 3A	0.81	0.79	0.85	0.77	**0.91**
Stage 3B	0.88	0.84	0.87	0.92	**0.94**
Stage 3C	0.80	0.80	0.81	0.91	**0.97**

**Table 4:** Accuracy values of machine learning methods used to predict whether a patient will die.

Models	All Features (29/29)	ANOVA (26/29)	CHI-2 (27/29)	KRUSKAL (27/29)
Fine Tree	0.76	0.76	0.74	0.76
Medium Tree	0.77	0.77	0.77	0.78
**Coarse Tree**	**0.81**	**0.81**	0.78	0.79
Linear Discriminant	0.79	0.78	0.78	0.78
Binary GLM Logistic Regression	0.77	0.78	0.77	0.77
Efficient Logistic Regression	0.76	0.78	0.78	0.78
Efficient Linear SVM	0.79	0.79	0.78	0.78
Kernel Naive Bayes	0.70	0.75	0.76	0.73
Linear SVM	0.78	0.80	0.79	0.78
Quadratic SVM	0.75	0.79	0.78	0.77
Cubic SVM	0.75	0.76	0.76	0.77
Fine Gaussian SVM	0.75	0.75	0.75	0.75
Medium Gaussian SVM	0.78	0.77	0.78	0.79
Coarse Gaussian SVM	0.75	0.75	0.75	0.75
Fine KNN	0.69	0.71	0.71	0.75
Medium KNN	0.77	0.77	0.77	0.78
Coarse KNN	0.75	0.75	0.75	0.75
Cosine KNN	0.74	0.75	0.76	0.78
Cubic KNN	0.75	0.78	0.74	0.77
Weighted KNN	0.76	0.77	0.77	0.77
Boosted Trees	0.79	0.79	0.80	0.79
**Bagged Trees**	0.80	0.79	**0.81**	**0.80**
Subspace Discriminant	0.77	0.78	0.78	0.78
Subspace KNN	0.77	0.79	0.78	0.77
RUSBoosted Trees	0.73	0.75	0.74	0.75
Narrow Neural Network	0.71	0.73	0.76	0.73
Medium Neural Network	0.71	0.73	0.73	0.76
Wide Neural Network	0.73	0.72	0.73	0.76
Bilayered Neural Network	0.72	0.70	0.71	0.72
Trilayered Neural Network	0.75	0.68	0.72	0.75
SVM Kernel	0.77	0.77	0.77	0.77
Logistic Regression Kernel	0.76	0.77	0.76	0.75

**Table 5:** Accuracy values of machine learning methods used to predict whether a patient will relapse.

Models	All Features (29/29)	ANOVA (26/29)	CHI2 (25/29)	KRUSKAL (22/29)
Fine Tree	0.81	0.80	0.78	0.77
Medium Tree	0.81	0.80	0.81	0.79
**Coarse Tree**	**0.84**	**0.82**	0.82	**0.83**
Linear Discriminant	0.80	0.81	0.81	0.82
Binary GLM Logistic Regression	0.81	0.81	0.82	0.82
Efficient Logistic Regression	0.81	0.81	0.81	0.82
**Efficient Linear SVM**	0.82	**0.82**	0.83	**0.83**
Kernel Naive Bayes	0.78	0.78	0.79	0.79
**Linear SVM**	0.81	0.81	0.82	**0.83**
**Quadratic SVM**	0.80	**0.82**	0.80	0.81
Cubic SVM	0.79	0.81	0.79	0.79
Fine Gaussian SVM	0.80	0.80	0.80	0.80
**Medium Gaussian SVM**	0.82	**0.82**	0.83	**0.83**
Coarse Gaussian SVM	0.80	0.80	0.80	0.80
Fine KNN	0.77	0.77	0.76	0.74
Medium KNN	0.79	0.81	0.80	0.80
Coarse KNN	0.80	0.80	0.80	0.80
Cosine KNN	0.80	0.80	0.81	0.79
Cubic KNN	0.80	0.81	0.81	0.80
Weighted KNN	0.79	0.80	0.79	0.79
**Boosted Trees**	0.81	**0.82**	**0.84**	**0.83**
**Bagged Trees**	0.82	**0.82**	0.82	0.82
Subspace Discriminant	0.80	0.79	0.79	0.80
Subspace KNN	0.78	0.79	0.81	0.80
RUSBoosted Trees	0.73	0.72	0.75	0.71
Narrow Neural Network	0.76	0.77	0.77	0.75
Medium Neural Network	0.76	0.77	0.75	0.79
Wide Neural Network	0.78	0.77	0.78	0.77
Bilayered Neural Network	0.78	0.77	0.75	0.75
Trilayered Neural Network	0.75	0.76	0.76	0.76
SVM Kernel	0.81	0.80	0.81	0.81
Logistic Regression Kernel	0.80	0.80	0.80	0.80

## Data Availability

The data supporting the findings of this study are available from the corresponding author upon reasonable request.
